# 

**Published:** 1876-03

**Authors:** 


[Vol. XV] MARCH, 1876. [No. 8.]

### BUFFALO

### Medical and Surgical Journal.

S. G. L. INDEXED

EDITED BY JULIUS F. MINER, M. D.

Professor of Special Surgery in the Buffalo Medical College ; Surgeon to the Buffalo General Hospital ; Surgeon to Buffalo Hospital of the Sisters of Charity, etc., etc.

AND

EDWARD N. BRUSH, M. D.

BUFFALO.
PUBLISHED FOR THE EDITORS.

1876.

